# 1366. Re-examining the Origins of Ebola virus Emergence

**DOI:** 10.1093/ofid/ofac492.1195

**Published:** 2022-12-15

**Authors:** Seth D Judson

**Affiliations:** Johns Hopkins University School of Medicine, Baltimore, Maryland

## Abstract

**Background:**

Ebola virus (EBOV) is one of four ebolaviruses known to cause Ebola virus disease (EVD). It is widely thought that EVD outbreaks originate from spillover of ebolaviruses from wildlife into humans. However, phylogenetic analysis of EBOV sequences from recent EVD cases reveal genetic similarity to EBOV from prior outbreaks. Therefore, it is likely that these recent EVD outbreaks originated through human-to-human transmission instead of wildlife spillover. The aim of this study is to re-examine the origins and contexts of EVD outbreaks given this new knowledge.

**Methods:**

All known EVD outbreaks and EBOV emergence events from 1976-2021 were analyzed via literature review. The primary and index cases for each outbreak were compared based on demographics and suspected sources of transmission. The diagnostic testing and treatment locations for each EVD index case were also investigated. Phylogenetic and epidemiologic relationships were examined to characterize whether outbreaks likely originated from separate spillover events or human-to-human transmission.

**Results:**

Overall, 22 outbreaks caused by EBOV were identified from 1976-2021 (Table). 5/22 (22.7%) of outbreaks were possibly linked to previous EVD outbreaks, including the four most recent outbreaks. Possible sources for these outbreaks included relapse and delayed sexual transmission from survivors. 12/22 (54.5%) of outbreaks were linked to suspected spillover sources, which included contact with duikers, chimpanzees, gorillas, monkeys, and bats. The locations of these Ebola virus emergence events are shown in the Figure.

**Figure:**
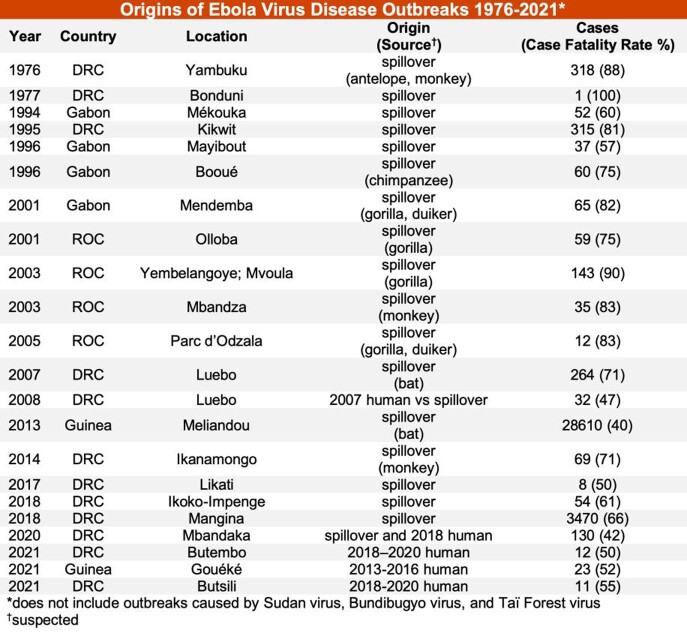

**Figure:**
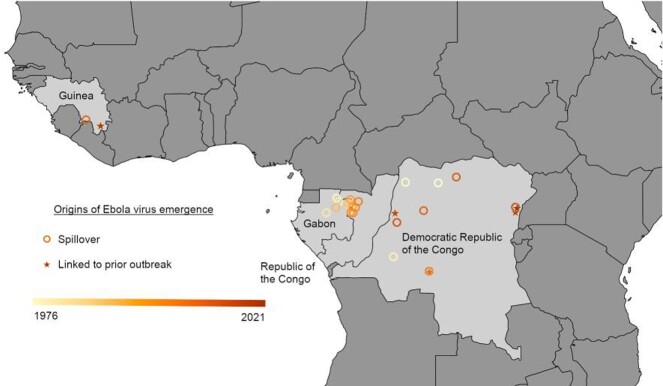

**Conclusion:**

Recent EVD outbreaks have changed our understanding of the emergence of EBOV in humans. Most recent outbreaks have originated from human-to-human transmission rather than spillover from wildlife. Multiple large EVD outbreaks have created the potential for future resurgence among humans. Therefore, increased surveillance among humans as well as awareness among healthcare workers and traditional healers, who are often the first to encounter index cases, will be critical to preventing the next epidemic. Clinicians and researchers will need to carefully evaluate the origins of EVD outbreaks through epidemiological and phylogenetic analyses while also preventing stigma among survivors.

**Disclosures:**

**All Authors**: No reported disclosures.

